# Establishment and validation of intravenous anesthesia with dexmedetomidine for pigs under assisted spontaneous breathing: A preclinical model of intensive care conditions

**DOI:** 10.1371/journal.pone.0293215

**Published:** 2023-10-18

**Authors:** Christin Wenzel, Sashko G. Spassov, Jörg Haberstroh, Johannes Spaeth, Stefan Schumann, Johannes Schmidt

**Affiliations:** 1 Department of Anesthesiology and Critical Care, Medical Center—University of Freiburg, Faculty of Medicine, University of Freiburg, Freiburg, Germany; 2 Experimental Surgery, Center for Experimental Models and Transgenic Service, Medical Center—University of Freiburg, Faculty of Medicine, University of Freiburg, Freiburg, Germany; Sapienza University of Rome: Universita degli Studi di Roma La Sapienza, ITALY

## Abstract

Large animal models are frequently used to investigate new medical approaches. In most cases, animals are kept under general anesthesia and mandatory mechanical ventilation during the experiments. However, in some situations assisted spontaneous breathing is essential, e.g. when simulating conditions in a modern intensive care unit. Therefore, we established an anesthesia regime with dexmedetomidine and midazolam/ketamine in porcine models of assisted spontaneous breathing. The total intravenous anesthesia was used in lung healthy pigs, in pigs with oleic acid induced acute respiratory distress syndrome and in pigs with methacholine induced bronchopulmonary obstruction. We were able to maintain stable conditions of assisted spontaneous breathing without impairment of hemodynamic, respiratory or blood gas variables in lung healthy pigs and pigs with induced acute respiratory distress syndrome for a period of five hours and in pigs with induced bronchopulmonary obstruction for three hours. Total intravenous anesthesia containing dexmedetomidine enables stable conditions of assisted spontaneous breathing in healthy pigs, in pigs with induced acute respiratory distress syndrome and in pigs induced bronchopulmonary obstruction as models of intensive care unit conditions.

## Introduction

Large animal models and particularly pigs are used regularly to investigate new medical methods and techniques, to study safety issues and potential treatment effects [1–5]. In this regard, it should be considered that animal models should reflect patient’s conditions as close as possible to clinical routine including anesthesia. Such animal models can be very complex including surgical interventions and manipulations. In most instances animals are under general anesthesia during the experiments, which usually entails the need for mandatory mechanical ventilation. Hemodynamic, respiratory and blood gas variables need to be kept in the physiological range for the whole experimental time to draw reliable conclusions of the respective study.

In certain clinical situations, e.g. on intensive care unit, patients are left to breath spontaneously or rather ventilation modes to assist spontaneous breathing are applied.

To reflect conditions of intensive care unit in animal models entails the use of assisted spontaneous breathing with appropriate anesthesia.

We aiming to establish and prove a new anesthesia regime of midazolam/ketamine augmented with dexmedetomidine in porcine models of assisted spontaneous breathing. For this purpose we investigated this anesthesia regime in lung healthy pigs, in pigs with oleic acid induced acute respiratory distress syndrome (ARDS) and in pigs with methacholine induced bronchopulmonary obstruction (OBST).

## Materials and methods

### Ethical approval

Animal experiments were reviewed, approved by the local authorities (Regierungspräsidium Freiburg, G-20/45) and performed in accordance to the European law on the protection of animals used for scientific purposes (EU-Directive 2010/63).

### Animal experiments

In total 15 German Landrace hybrid pigs of both sex with a weight of 48±6 kg were included in this study. Pigs were assigned to one of the following groups: healthy pigs (Healthy, n = 5), pigs with oleic acid induced ARDS (n = 5) or pigs with methacholine induced OBST (n = 5).

After sedation with an intramuscular injection of midazolam (0.5 mg∙kg^-1^; Rotexmedica GmbH, Trittau, Germany) and ketamine (20 mg∙kg^-1^; Serumwerk Bernburg AG, Bernburg, Germany), pigs were placed in supine position on a heating pad to maintain physiological porcine body temperature (37–38°C). An intravenous line was placed in the left ear vein for total intravenous anesthesia (TIVA) and infusion of crystalloid fluid (10 ml∙kg^-1^∙h^-1^; Ringer-Infusionslösung B. Braun, B. Braun Melsungen AG, Melsungen, Germany). To allow tracheal intubation with an endotracheal tube (7.5 mm Shiley, Covidien, Mansfield, MA), anesthesia was induced with intravenous administration of propofol (single treatment as needed; Propofol 10 mg∙ml^-1^, Fresenius Kabi Deutschland GmbH, Bad Homburg; Germany).

Before placement of catheters, pigs received a single bolus of 0.005 mg∙kg^-1^ fentanyl (Fentanyl 0.05 mg∙ml^-1^, Rotexmedica GmbH, Trittau, Germany). A pulmonary artery catheter (7F, Edwards Lifescience, Irvine, CA) was inserted in the right internal jugular vein via an introducer sheath (8.5F, Arrow, Teleflex Medical GmbH, Fellbach, Germany). An arterial catheter (4F, Pulsion Medical Systems, Feldkirchen, Germany) was placed in the femoral artery for blood pressure measurement and arterial blood sampling for blood gas analyses (ABL90FLEX BGA analyser, Radiometer, Krefeld, Germany). A suprapubic urinary catheter (12F, Uromed Kurt Drews KG, Oststeinbek, Germany) was inserted in the bladder. Catheterization were performed under mechanical ventilation (VC-SIMV, EVITA V500, Dräger, Lübeck, Germany) with a tidal volume (V_T_) of 7 ml∙kg^-1^, inspiratory oxygen fraction (FiO_2_) of 0.3 and a positive end-expiratory pressure (PEEP) of 5 cmH_2_O. Anesthesia was maintained during the experiment using continuous intravenous infusion of 0.8–1.5 mg∙kg^-1^∙h^-1^ midazolam (Midazolam-hameln 2mg∙ml^-1^, Hameln Pharma GmbH, Hameln; Germany), 3–5 mg∙kg^-1^∙h^-1^ ketamine and 4–6 μg∙kg^-1^∙h^-1^ dexmedetomidine (10 μg/ml, Orion Corporation, Espoo, Finland).

After surgical preparation the animals were pre oxygenated under volume controlled mechanical ventilation for 5 min with a FiO_2_ = 0.7. Then the ventilation mode was switched to assisted spontaneous breathing with volume support (SPN-CPAP/VS, EVITA V500, Dräger, Lübeck, Germany), followed by a stabilization period of 10 min.

In pigs with healthy lungs assisted spontaneous breathing with a target V_T_ of 7 ml∙kg^-1^ was maintained for five hours after the stabilization period. FiO_2_ was set to 0.3 and PEEP was 5 cmH_2_O. Trigger sensitivity was set to 4 l∙min^-1^.

In pigs with oleic acid induced ARDS, PEEP was set to 10 cmH_2_O, FiO_2_ = 0.7 and trigger sensitivity to 1 l∙min^-1^ before induction of the lung injury. Induction of lung injury was performed as described [2]. In brief, oleic acid was infused continuously via the intravenous port (10 ml∙h^-1^) until stable ARDS conditions. These conditions were defined by a PaO_2_/FiO_2_-ratio between 90 and 160 mmHg. Observation time of five hours started, after a stabilization period of 20 min.

For pigs with methacholine induced OBST the PEEP was set to 5 cmH_2_O and trigger sensitivity was set to 2 l∙min^-1^. Subsequently OBST was induced as described [6]. In brief, nebulized (Aerogen Pro-X (Solo), INSPIRATION Medical GmbH, Bochum, Germany) methacholine (0.2 mg∙kg^-1^, Cayman Chemical Company, Ann Arbor, MI) was (Aerogen Pro-X (Solo), INSPIRATION Medical GmbH, Bochum, Germany) administered via the breathing circuit until a stable OBST was achieved. Stable OBST was defined by doubling of the airway resistance, as read from the ventilator. After induction of stable OBST, observation period of three hours started. The period of three hours was defined, due to the limited effect duration of methacholine.

### Measurements and data processing

**Fig 1:**
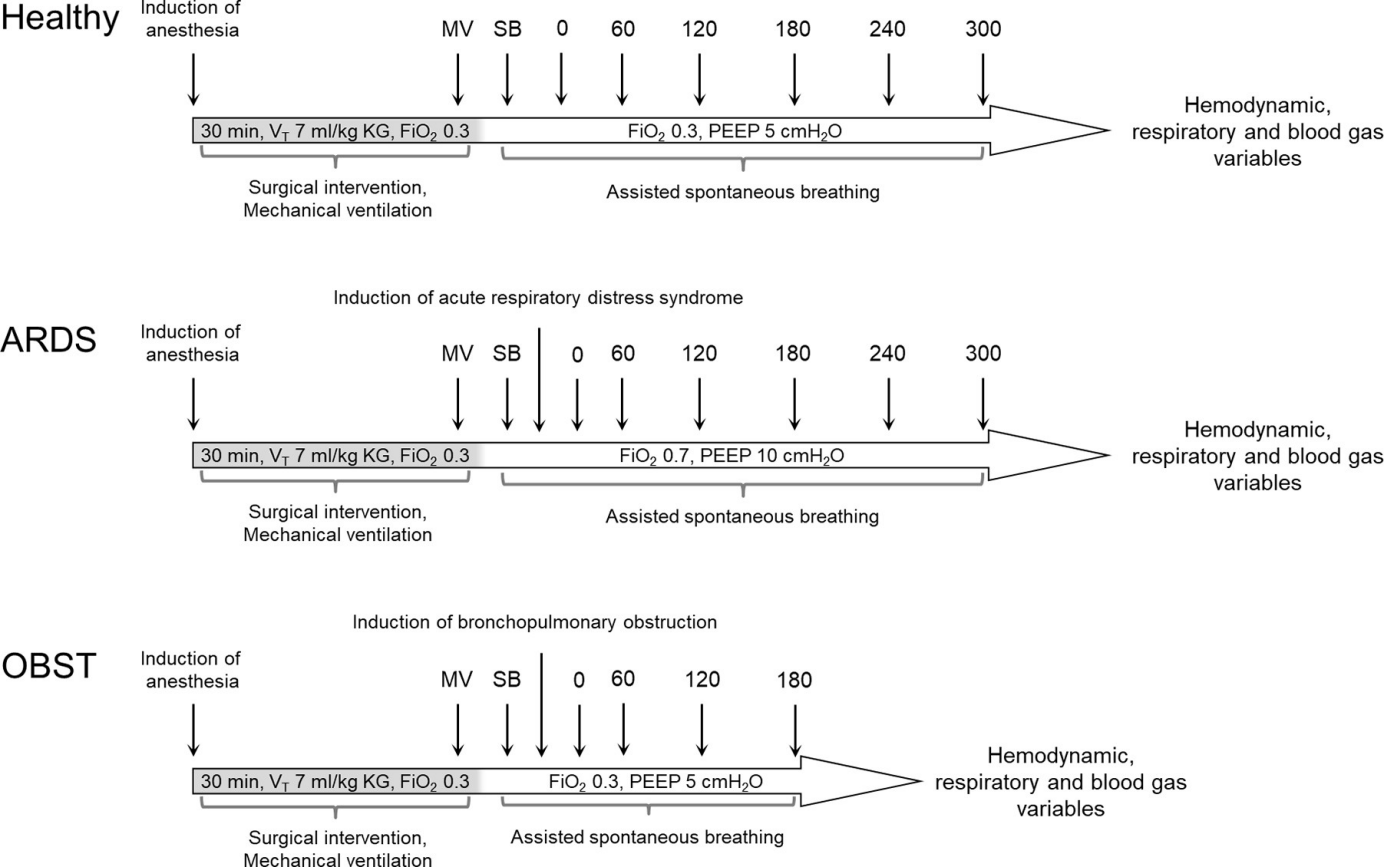
Timeline of the experiments. Measurement times of the experiments for pigs with healthy lungs (Healthy), the model of acute respiratory distress syndrome (ARDS) and the model of bronchopulmonary obstruction (OBST). Hemodynamic, respiratory as well as blood gas variables were documented 10 min after surgical interventions during mandatory ventilation (MV) and after switching ventilation mode to assisted spontaneous breathing with a stabilization period of 10 min (SB). Further data documentations were performed after induction of stable acute respiratory distress syndrome with oleic acid or bronchopulmonary obstruction with methacholine (0), respectively. Afterwards data were documented every hour (60, 120, 180, 240 and 300). Observation time for Healthy and ARDS was 5 h. Observation time for OBST was 3 h. FiO_2_: inspiratory oxygen fraction, PEEP: positive end-expiratory pressure.

Variables of hemodynamic, respiratory and blood gas analyses were documented for the following times: 10 min after the surgical intervention during mandatory mechanical ventilation (MV) and 10 min after switching the ventilation mode to assisted spontaneous breathing (SB). In the ARDS model and the OBST model another data documentation was performed after induction of the respective condition (time 0). Afterwards data were documented hourly (Fig 1).

The duration of apnea after switching from mandatory ventilation to assisted spontaneous breathing was determined as the time between the last mandatory breath and the first spontaneously triggered breath. Hemodynamic, respiratory and blood gas variables during mechanical ventilation were compared to those during assisted spontaneous breathing. Additionally, time courses of hemodynamic, respiratory and blood gas variables were analyzed over the observation time for each model, respectively. All interventions (placement of catheters and subsequent experimental time) were performed under deep anesthesia to alleviate suffering. To verify anesthesia depth, we checked electrocardiogram, heart rate, blood pressure. Furthermore, we had a look at eyelid, cornea and pain reflexes of the animal in regular manner. At the end of the experiment, animals were killed under deep anesthesia with overdose of anesthesia and 1 M potassium chloride solution (1 ml/kg, Fresenius Kabi Deutschland GmbH, Bad Homburg, Germany).

### Statistical analysis

Data are presented as mean±SD or box plots showing individual values, median, upper and lower quartile and minimal and maximal values. The time-dependent changes of the recorded variables between mechanical ventilation and assisted spontaneous breathing within the groups (including all animals n = 15) were compared using paired t-test (GraphPad PRISM, Ver. 8.1.2, GraphPad Software Inc., La Jolla, CA). For comparison of the time course of hemodynamic, respiratory and blood gas variables repeated measure mixed-effects analysis were performed followed by Tukey’s multiple comparison test if the mixed-effects analysis indicated a significant influence of the time points. A P<0.05 was considered significant.

## Results

### Switch from mandatory ventilation to assisted spontaneous breathing

**Fig 2:**
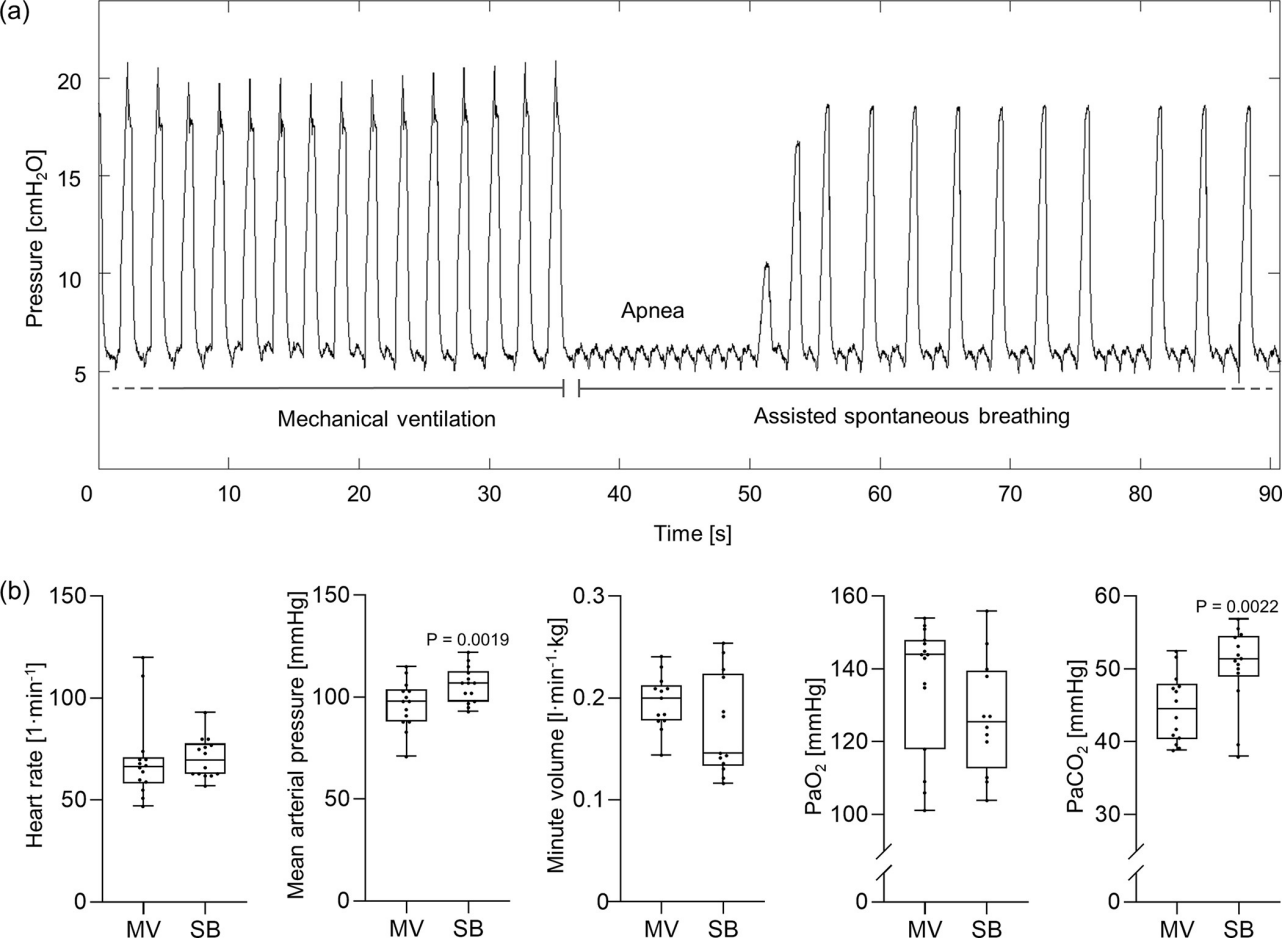
Exemplary airway pressure curve over time. (a) Switch from mandatory ventilation to assisted spontaneous breathing and (b) variables of hemodynamic, respiratory system and blood gas analyses. The box plots show individual values, median (n = 15), upper and lower quartile and minimal and maximal values (whiskers). Paired t-test; PaCO_2_: arterial partial pressure of carbon dioxide; PaO_2_: arterial partial pressure of oxygen.

Ventilation was sufficient after switching from mandatory ventilation to assisted spontaneous breathing in all groups. The switching from mandatory ventilation to assisted spontaneous breathing mode was associated with a brief apnea phase (Fig 2A) of 6 s (range between 2 to 14 s). Heart rate, minute volume, arterial partial oxygen pressure (PaO_2_), oxygen saturation (SpO_2_), bicarbonate, base access, hemoglobin and lactate were comparable during mandatory ventilation and assisted spontaneous breathing (Fig 2B, Table 1). Whereas, mean arterial pressure (P = 0.0019) and arterial partial carbon dioxide pressure (PaCO_2_, P = 0.0022) were higher and pH (P = 0.0048) was lower during assisted spontaneous breathing compared to mandatory ventilation.

**Table 1:** Blood gas variables for pigs during mandatory ventilation and after switching ventilation mode to assisted spontaneous breathing.

Measurement	Mandatory ventilation	Assisted spontaneous breathing	P value
**Variables**			
**SpO**_**2**_ [%]	100±1	100±1	0.8364
**pH**	7.438±0.046	7.394±0.055 *	0.0048
**HCO**_**3**_^**-**^ [mmol∙l^-1^]	29.7±1.4	30.1±2.1	0.3683
**BE** [mmol∙l^-1^]	5.8±1.8	6.0±1.9	0.5190
**Hb** [g∙dl^-1^]	9.7±0.9	9.6±0.9	0.5842
**Lactate** [mmol∙l^-1^]	1.1±0.4	1.2±1.0	0.6618

All animals (n = 15) were included in this analysis. Paired t-test was performed

*: compared to mandatory ventilation. BE: base excess, Hb: hemoglobin, HCO_3_^-^: bicarbonate concentration; SpO_2_: oxygen saturation.

### Assisted spontaneous breathing in healthy pigs

Mixed-effects analysis showed time dependent differences for mean arterial pressure (P = 0.0145), minute volume (P = 0.0440) and hemoglobin (P = 0.0316), Table 2. Mean arterial pressure showed differences between 120 and 180 *vs*. 60 (P = 0.0184 and P = 0.0426), hemoglobin between 180 and 300 *vs*. 0 (P = 0.0248 and P = 0.0104) and 300 *vs*. 240 (P = 0.0223).

**Table 2:** Hemodynamic, respiratory and blood gas variables of pigs with healthy lungs.

Time	0 min	60 min	120 min	180 min	240 min	300 min
**Variables**						
**HR** [1∙min^-1^]	68±7	81±9	80±11	82±9	81±8	80±6
**MAP** [mmHg] *	98±14	103±11	107±11 ^$^	109±11 ^$^	115±15	111±12
**AMV** [l∙min^-1^∙kg^-1^] *	0.14±0.03	0.17±0.02	0.17±0.02	0.16±0.02	0.16±0.01	0.15±0.01
**SpO**_**2**_ [%]	100±0	100±1	100±0	100±0	100±1	100±0
**PaO**_**2**_ [mmHg]	125±21	135±12	137±10	130±12	129±8	128±6
**PaCO**_**2**_ [mmHg]	52±4	49±3	48±2	48±1	48±1	48±1
**pH**	7.375±0.056	7.422±0.016	7.429±0.014	7.427±0.014	7.429±0.014	7.432±0.015
**HCO**_**3**_^**-**^ [mmol∙l^-1^]	31.7±1.6	31.3±1.5	31.1±1.4	30.9±1.4	31.2±1.4	31.2±1.2
**BE** [mmol∙l^-1^]	6.3±1.7	6.8±1.6	6.8±1.6	6.6±1.7	7.0±1.8	7.0±1.8
**Hb** [g∙dl^-1^] *	9.0±0.6	9.5±0.8	9.4±0.9	9.6±0.8 ^#^	9.7±0.8	10±0.8 ^#^ ^ß^
**Lactate** [mmol∙l^-1^]	0.9±0.2	1.0±0.2	1.0±0.2	0.9±0.1	0.8±0.2	0.7±0.3

All n = 5.

*: Mixed-effects analysis showed changes over time

#: P<0.05 to 0 min

$: P<0.05 to 60 min

ß: P<0.05 to 240 min.

AMV: minute volume; BE: base excess; Hb: hemoglobin; HCO_3_^-^: bicarbonate concentration; HR: heart rate; MAP: mean arterial pressure; PaCO_2_; arterial partial pressure of carbon dioxide; PaO_2_: arterial partial pressure of oxygen; SpO_2_: oxygen saturation; 0, 60, 120, 180, 240, 300: measurement time points 0, 60, 120, 180, 240 and 300 min after establishment of assisted spontaneous breathing.

### Assisted spontaneous breathing in ARDS

The induction of acute respiratory distress syndrome resulted in a decrease of PaO_2_/FiO_2_ from 440±63 mmHg to 124±26 mmHg and was subsequent stable over the observation period. Heart rate (P = 0.0361) and minute volume (P = 0.0395) increased over observation period in ARDS pigs, while mean arterial pressure, SpO_2_ and blood gas variables did not show differences over time (Table 3).

**Table 3:** Hemodynamic, respiratory system and blood gas variables of pigs with induced acute respiratory distress syndrome.

Time	0 min	60 min	120 min	180 min	240 min	300 min
**Variables**						
**HR** [1∙min^-1^] *	69±16	81±24	98±40	104±33	106±29	110±22
**MAP** [mmHg]	101±19	101±13	99±22	97±17	89±19	83±20
**AMV** [l∙min^-1^∙kg^-1^] *	0.22±0.04	0.27±0.04	0.28±0.06	0.29±0.05	0.29±0.05	0.31±0.06
**SpO**_**2**_ [%]	97±2	98±3	97±5	97±4	96±5	93±8
**PaO**_**2**_ [mmHg]	87±19	90±16	81±15	81±18	77±24	75±20
**PaCO**_**2**_ [mmHg]	50±6	48±4	49±4	47±3	48±3	47±2
**pH**	7.378±0.043	7.394±0.021	7.385±0.029	7.391±0.026	7.388±0.031	7.385±0.031
**HCO**_**3**_^**-**^ [mmol∙l^-1^]	27.4±0.5	27.4±0.7	27.2±0.7	27.1±0.9	26.9±1.2	26.6±1.4
**BE** [mmol∙l^-1^]	4.2±0.4	4.0±1.1	4.0±0.8	3.8±1.1	3.5±1.4	3.1±1.6
**Hb** [g∙dl^-1^]	11.4±0.8	11.4±0.4	11.5±0.6	11.6±0.5	11.6±0.5	11.6±0.7
**Lactate** [mmol∙l^-1^]	1.6±0.7	1.3±0.4	1.1±0.2	1.0±0.1	1.1±0.1	1.1±0.1

All n = 5.

*: Mixed-effects analysis showed changes over time (P<0.05).AMV: minute volume; BE: base excess; Hb: hemoglobin; HCO_3_^-^: bicarbonate concentration; HR: heart rate; MAP: mean arterial pressure; PaCO_2_: arterial partial pressure of carbon dioxide; PaO_2_: arterial partial pressure of oxygen; SpO_2_: oxygen saturation; 0, 60, 120, 180, 240, 300: measurement time points 0, 60, 120, 180, 240 and 300 min after induction of stable ARDS.

### Assisted spontaneous breathing during bronchopulmonary obstruction

Induction of OBST resulted in increase of resistance from 9±1 cmH_2_O∙l^-1^∙s^-1^ to 19±2 cmH_2_O∙l^-1^∙s^-1^ and was subsequently stable over the observation time (Table 4). In pigs with induced OBST no time dependent changes in hemodynamic, respiratory or blood gas variables were determined.

**Table 4:** Hemodynamic, respiratory system and blood gas variables of pigs with induced stable bronchopulmonary obstruction.

Time	0 min	60 min	120 min	180 min
**Variables**				
**HR** [1∙min^-1^]	75±10	81±8	81±5	85±10
**MAP** [mmHg]	119±11	118±9	123±9	119±11
**AMV** [l∙min^-1^∙kg^-1^]	0.1±0.1	0.1±0.1	0.1±0.1	0.1±0.1
**SpO**_**2**_ [%]	96±4	97±4	98±3	98±4
**R** [cmH_2_O∙l^-1^∙s^-1^]	19±2	18±2	18±3	16±3
**PaO**_**2**_ [mmHg]	80±17	83±17	90±18	92±23
**PaCO**_**2**_ [mmHg]	47±1	50±8	46±2	47±3
**pH**	7.421±0.034	7.429±0.024	7.430±0.028	7.426±0.039
**HCO**_**3**_^**-**^ [mmol∙l^-1^]	29.3±2.3	29.9±2.0	29.6±1.7	29.5±1.6
**BE** [mmol∙l^-1^]	6.0±2.5	6.5±2.1	6.3±1.9	6.2±1.7
**Hb** [g∙dl^-1^]	10.4±0.7	10.3±0.4	10.2±0.5	10.4±0.5
**Lactate** [mmol∙l^-1^]	0.7±0.2	0.7±0.2	0.7±0.2	0.7±0.2

All n = 5. AMV: minute volume; BE: base excess; Hb: hemoglobin; HCO_3_^-^: bicarbonate concentration; HR: heart rate; MAP: mean arterial pressure; MV: minute volume; PaCO_2_: arterial partial pressure of carbon dioxide; PaO_2_: arterial partial pressure of oxygen; SpO_2_: oxygen saturation; 0, 60, 120, 180: measurement time points 0, 60, 120 and 180 min after induction of stable bronchopulmonary obstruction; R: resistance.

## Discussion

We established a total intravenous anesthesia regime based on dexmedetomidine in combination with midazolam/ketamine to provide a possibility for assisted spontaneous breathing in pig models. Thereby, assisted spontaneous breathing was sufficient in pigs with healthy lungs as well as in pig models of acute respiratory distress syndrome or bronchopulmonary obstruction.

Often intravenous anesthesia is associated with hemodynamic impairment and especially reduction of blood pressure, usually associated with the administration of propofol and / or opioids. Furthermore, propofol and opioids reduce the respiratory drive. By contrast, in the study presented here, hemodynamic, respiratory and blood gas variables were stable or improved slightly over a medium-term observation period without causing respiratory depression. Pigs triggered the ventilator reliably over the observation periods with only a short apnea phase after switching from mandatory ventilation to assisted spontaneous breathing. Blood pressure was stable in a physiological range over the whole observation period. This is probably on behalf of the agonistic effect of dexmedetomidine on alpha-adrenoceptors, triggering vasoconstriction and resulting in an elevated blood pressure [7]. This was valid for healthy pigs as well as for pigs with induced OBST. The animals with induced ARDS showed a rather declining mean arterial pressure and an increasing heart rate, whereas only the change of the heart rate was statistically significant. This may be explained by the underlying lung damage. The oleic acid causes a damage to the lung capillaries, which in turn leads to a collapse of the microstructure of the alveoli. In addition to the obvious effect of an impaired gas exchange, this leads via an abrupt increase of the pulmonary artery pressure to right heart failure. In consequence, the left ventricle is impaired as well, which is expressed by the increased heart rate and the decreased arterial pressure. The clinical treatment of the right heart failure would include a differential therapy with inotropic substances, goal directed fluid administration and reduction of the pulmonary arterial vascular resistance [8]. This complex treatment was not part of this study.

Dexmedetomidine combines sedative and analgesic effects without respiratory depression [7, 9] compared to conventional anesthesia with propofol or opioids, which are associated with respiratory depression [10, 11]. Dexmedetomidine is well established for sedation in humans in the intensive care unit [7, 9]. By contrast, little is known about the effects of dexmedetomidine in pigs [10]. Pig models are established and accepted as pre-clinical model [1–4, 12–14]. In general, new medical approaches, techniques or anesthesia regimes need to be tested in pre-clinical studies before clinical application. In the study presented here, we reversed this approach and used an established anesthetic for humans to open a new opportunity to perform research in animal models. Thus, the presented anesthesia regime opens an additional opportunity to generate conditions close to clinical routine.

To the authors’ best knowledge, this is the first study presenting TIVA for spontaneous breathing in pigs without impairment of hemodynamic, respiratory and blood gas variables for five hours. Previous studies show the potential of anesthesia using dexmedetomidine as one substance of anesthetics’ combination for short-term anesthesia up to two hours e.g. for transport or non-surgical interventions. Authors reported that hemodynamic variables were stable and breathing patterns were regular [15, 16]. This is in accordance with the results presented here. Another study investigated the potential of TIVA using dexmedetomidine to maintain anesthesia with a duration of 12–24 h for pigs being ventilated mandatorily [11]. Additionally, a superiority of anesthesia with dexmedetomidine regarding cardiovascular response during mechanical ventilation in healthy pigs was reported compared to anesthesia with fentanyl [10]. Based on the results of our study we could extend the scope of dexmedetomidine to assisted spontaneous breathing in healthy pigs as well as in pigs with lung impairment. This included also surgical interventions and induction of lung injury. Of note, the experiments were never terminated for reasons of the anesthesia regime but according to the protocol.

Spontaneous breathing is essential in some clinical situations, even for treatment of ARDS on intensive care unit [17]. Therefore, it is a requirement to have animal models in accordance with clinical routine for further research.

The presented study was not designed to compare the different models (Healthy, ARDS and OBST), we rather aimed to present a possibility to perform porcine animal models with assisted spontaneous breathing and surgical interventions.

## Conclusion

TIVA with dexmedetomidine and midazolam/ketamine enables sufficient assisted spontaneous breathing in lung healthy pigs as well as pigs with induced ARDS or bronchopulmonary obstruction as models of intensive care unit conditions. Hemodynamic, respiratory and blood gas variables are not impaired.
